# Supplementary material for: IL-21, Inflammatory Cytokines and Hyperpolarized CD8+ T Cells Are Central Players in Lupus Immune Pathology
Source: Antioxidants (Basel). 2023 Jan 12;12(1):181. doi: 10.3390/antiox12010181 (PMC9855022; doi:10.3390/antiox12010181)
Supplement: Supplementary file 1 [file antioxidants-12-00181-s001.zip › SUPPLEMENTARY FIGS.pdf]

Figure S1

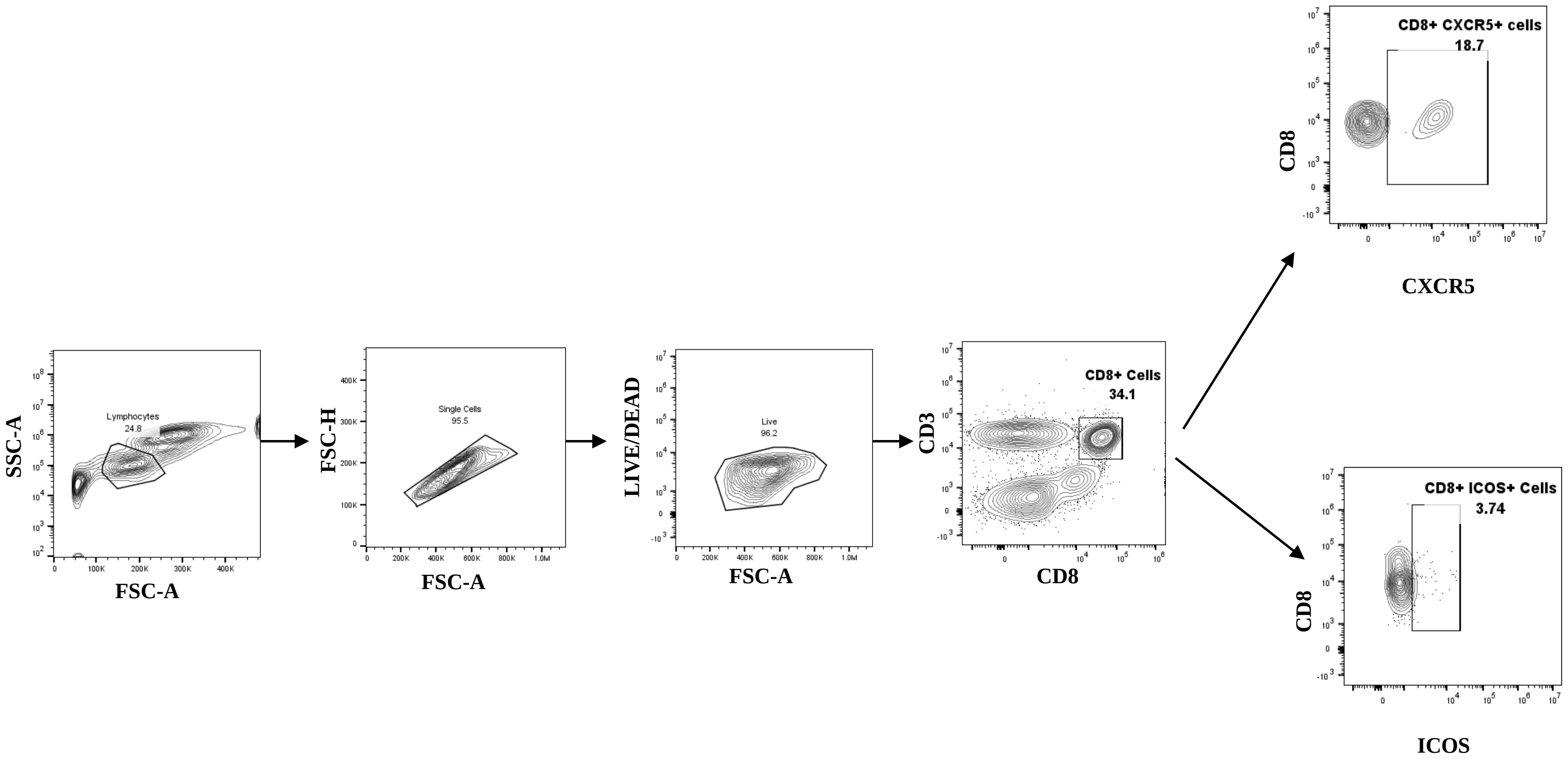

Figure S1.Gating strategy to assess surface markers from Whole blood of SLE patients and healthy controls.

Figure S2a

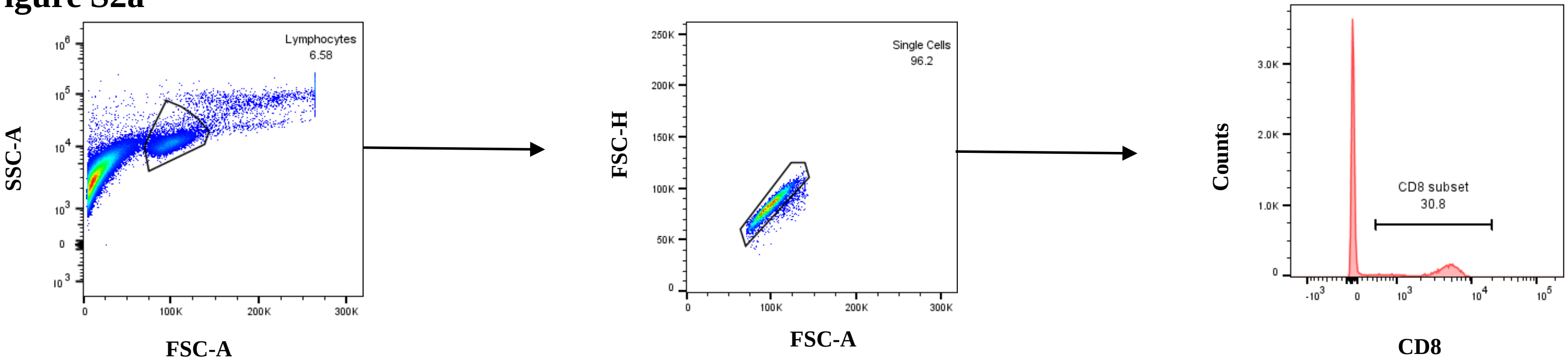

Figure S2b

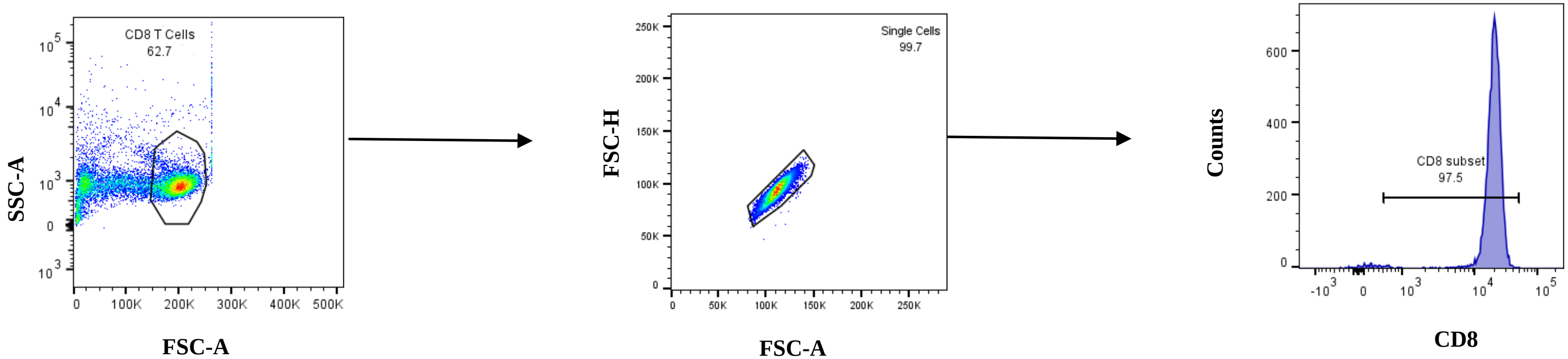

Figure S2c

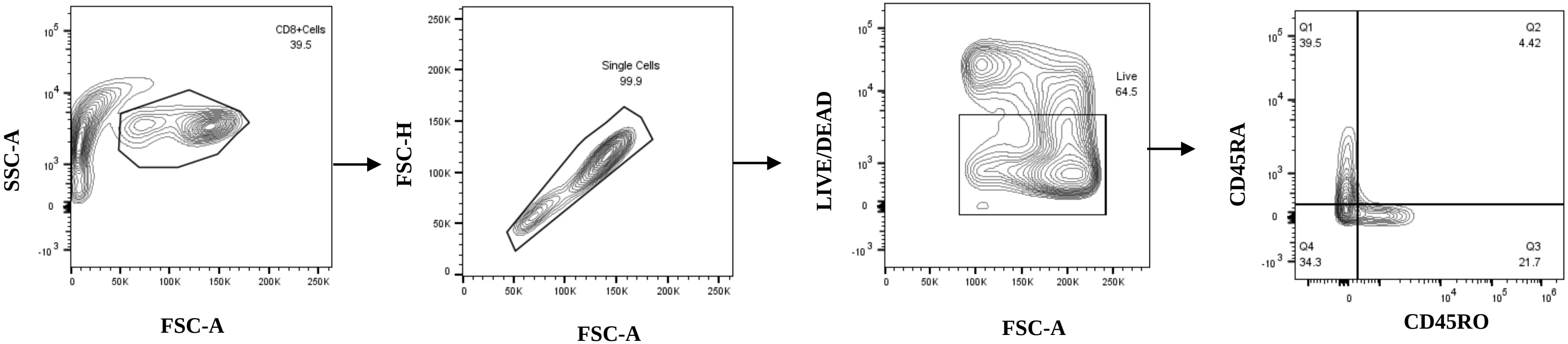

Figure S2a. Percentage of CD8+ T cells in PBMC of SLE patients. Figure S2b. percentage of purity of CD8+ T cells after isolation.

Figure S2c. Gating strategy to determine naïve, effector and memory compartments of CD8+ T cells.

Figure S3

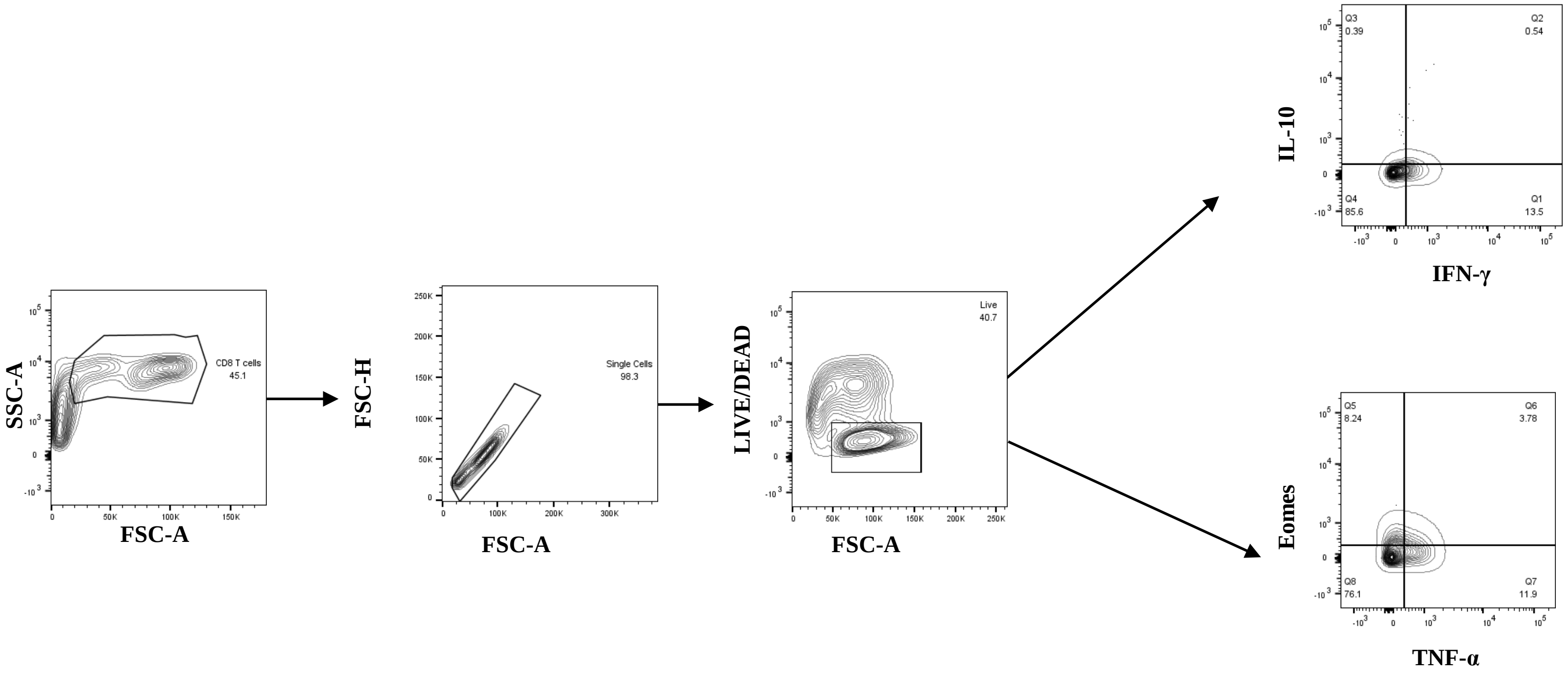

Figure S3. Gating strategy to determine transcription factor and cytokines staining from CD8+ cells of SLE Patients and healthy controls.

Figure S4

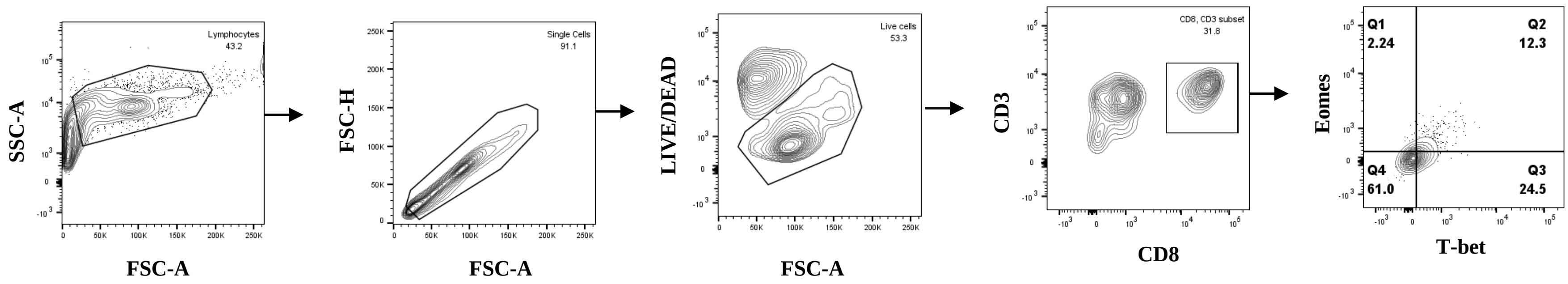

Figure S4. Gating strategy to determine transcription factors from both SLE and healthy controls from PBMCS.

Figure S5

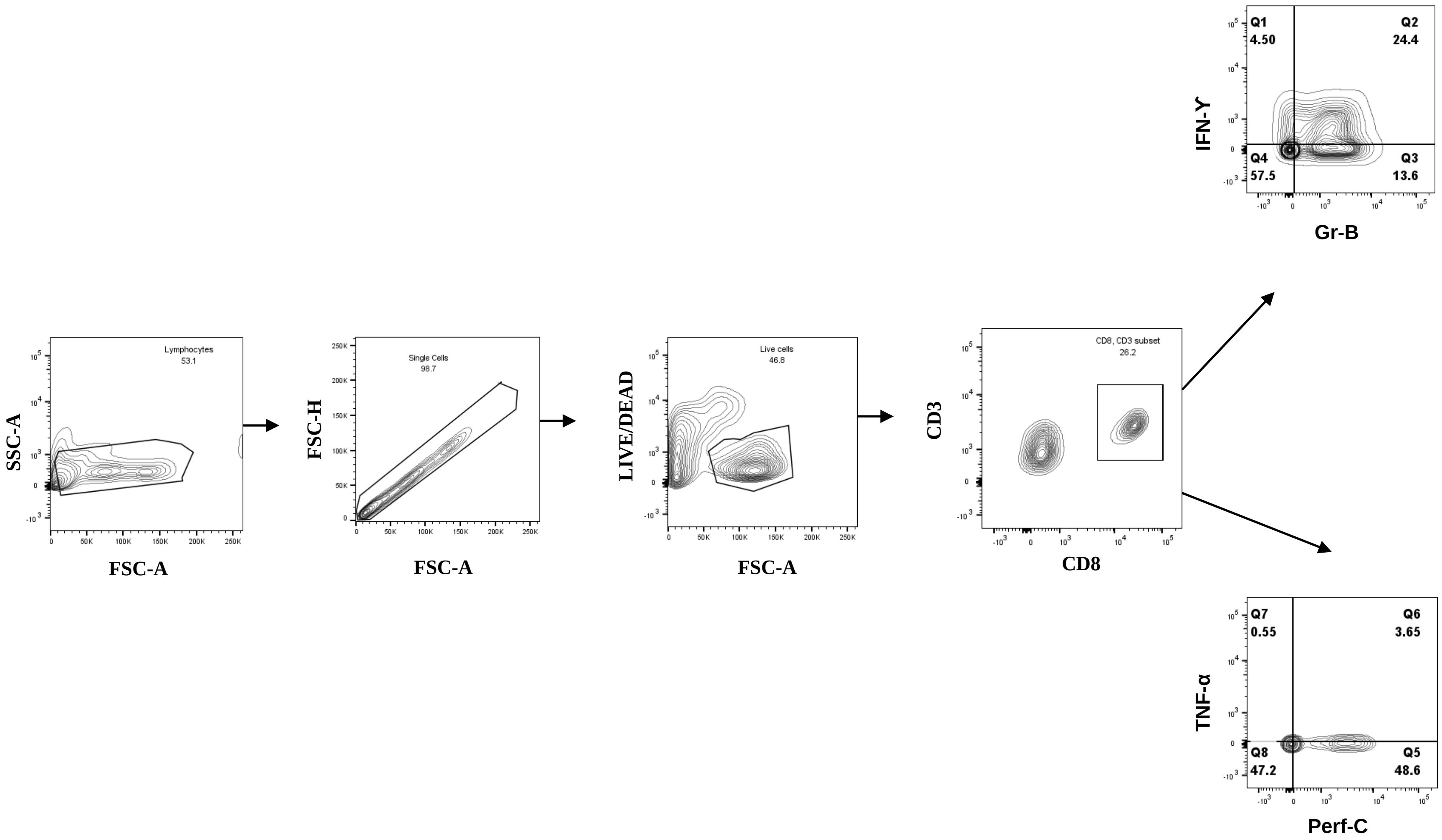

Figure S5. Gating strategy to determine cytotoxic and inflammatory cytokines from both SLE and healthy controls from PBMCS.

Figure S6a

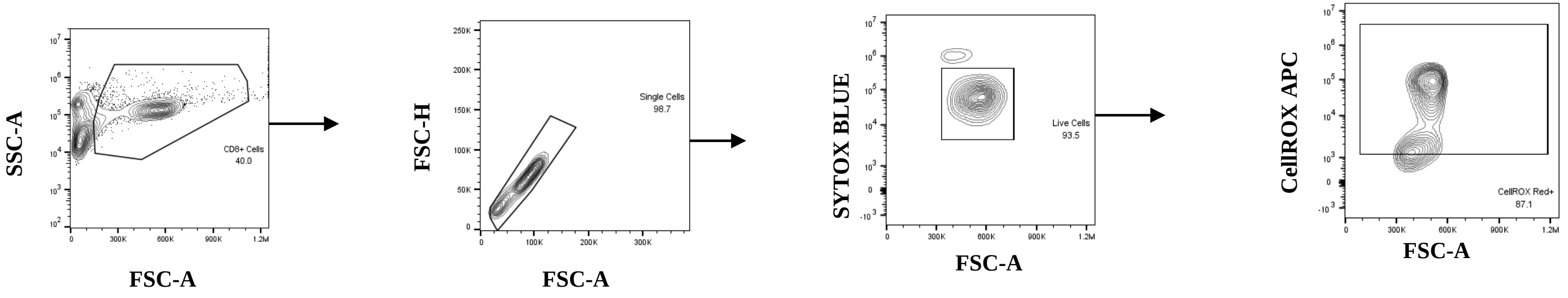

Figure S6b

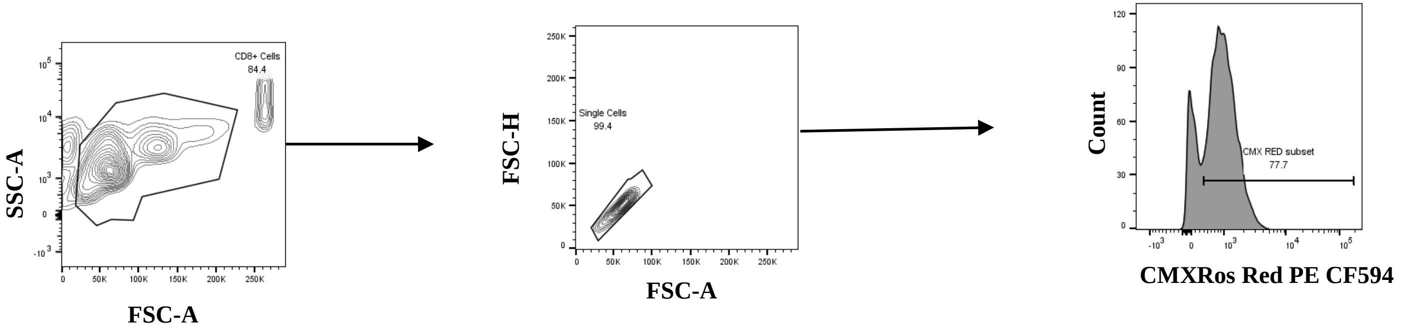

Figure S6a. Gating strategy to determine cellular ROS in CD8+ T cells of both SLE patients and healthy controls.
Figure S6b. Gating strategy to determine mitochondrial hyperpolarization in CD8+ T cells of SLE patients and healthy controls.

Figure S7

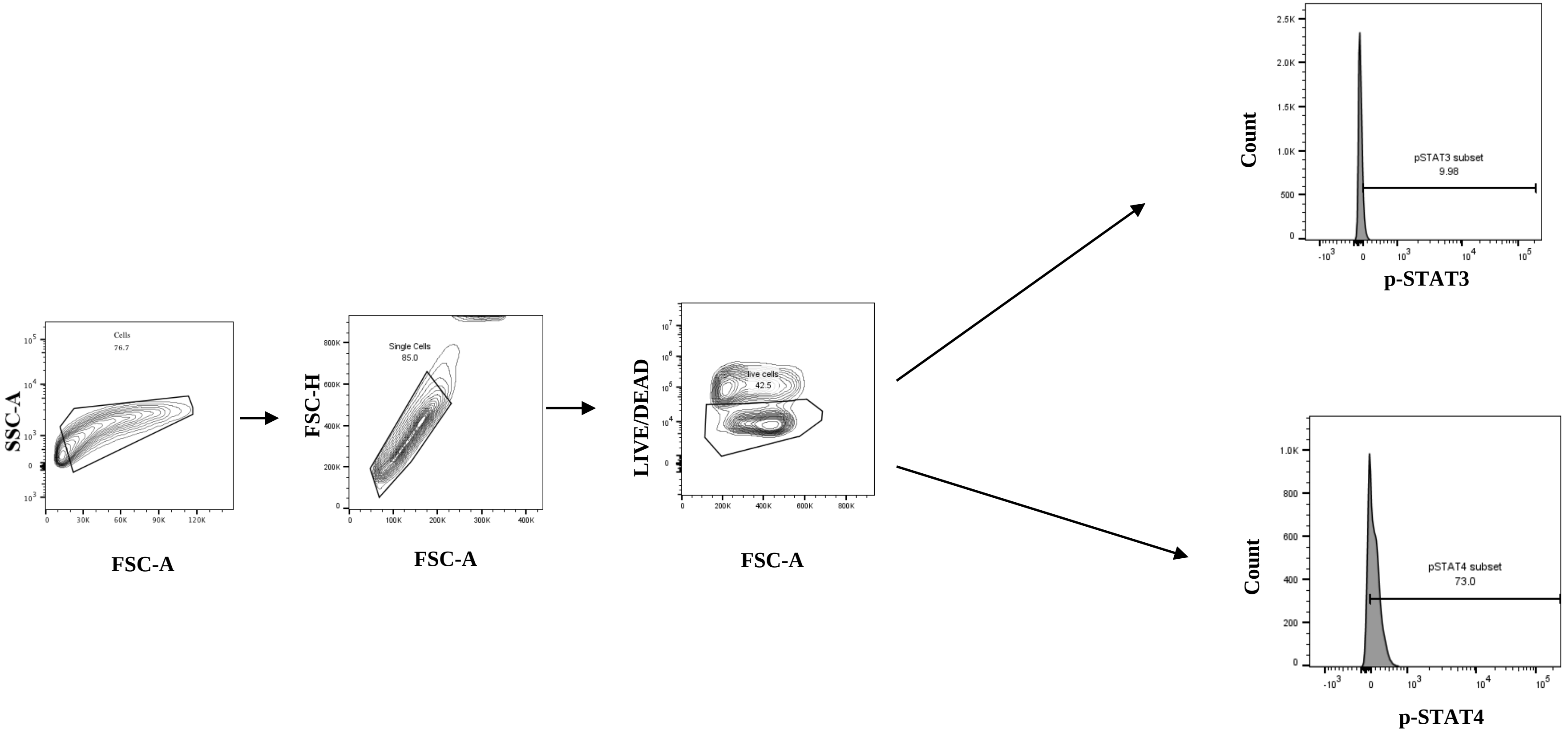

Figure S7. Gating strategy to determine phospho -STAT proteins from CD8+ T cells of SLE patients and healthy controls.

Figure S8

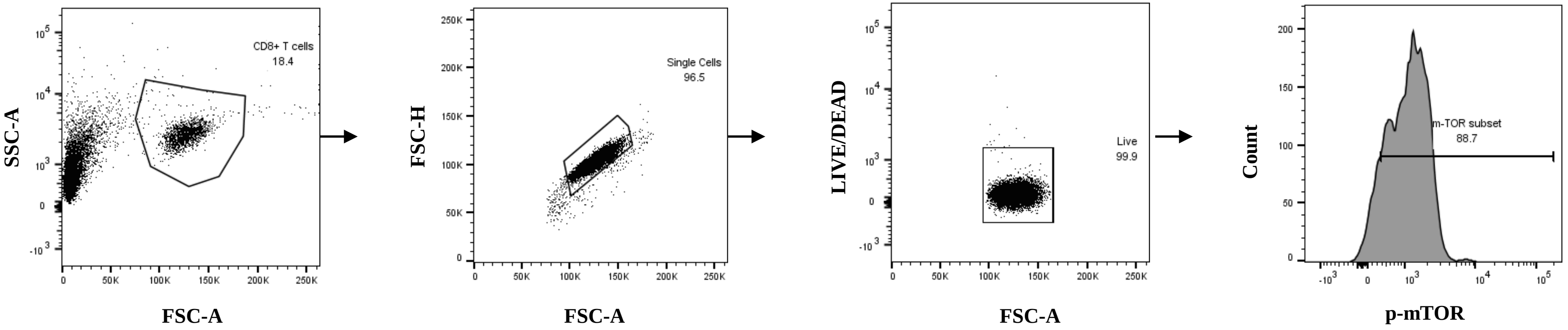

Figure S8 . Gating strategy to determine phospho mTOR proteins from CD8+ T cells of SLE patients .

Figure S9

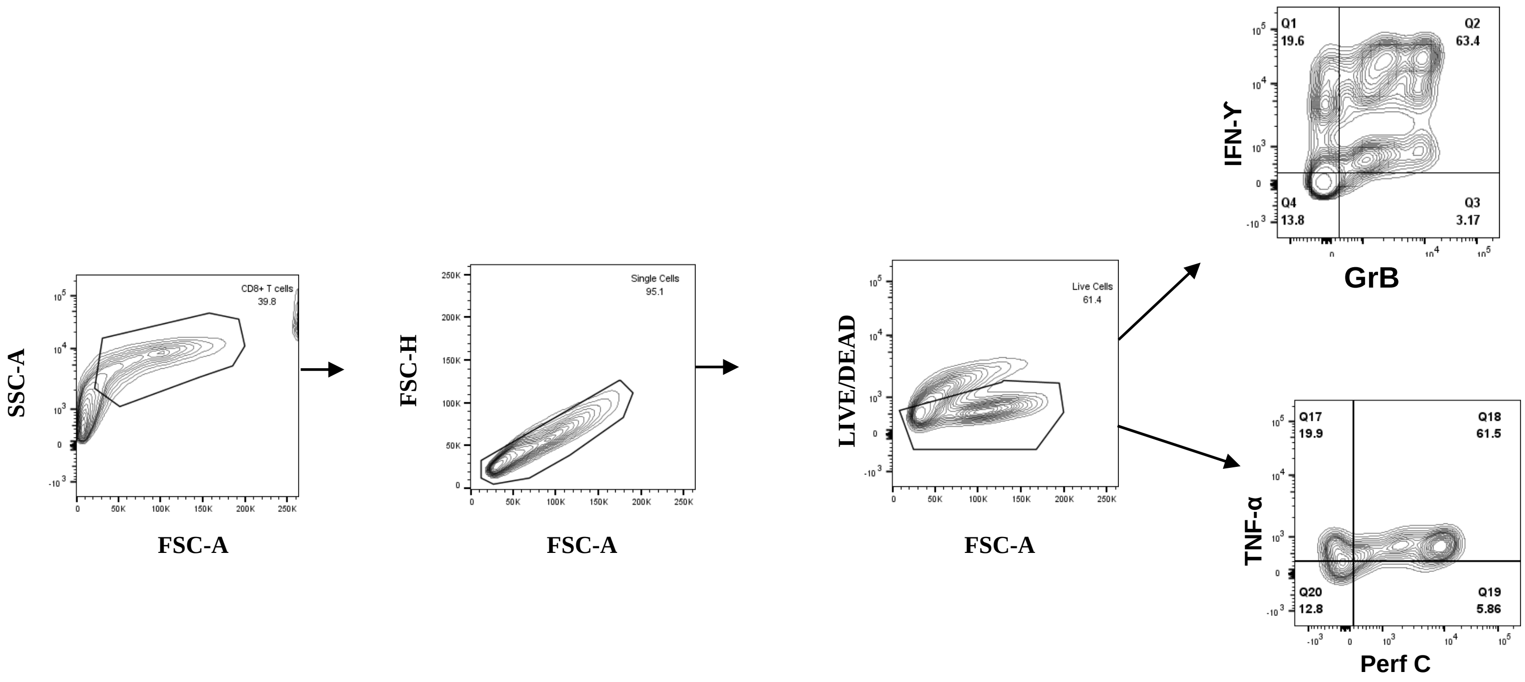

Figure S9. Gating strategy to determine Cytotoxic and inflammatory molecules from Tc1 and Tc21 Cells.
