# Supplementary material for: IL-21, Inflammatory Cytokines and Hyperpolarized CD8+ T Cells Are Central Players in Lupus Immune Pathology
Source: Antioxidants (Basel). 2023 Jan 12;12(1):181. doi: 10.3390/antiox12010181 (PMC9855022; doi:10.3390/antiox12010181)
Supplement: Supplementary file 1 [file antioxidants-12-00181-s001.zip › Table_S1 Reagents and Software used.pdf]

REAGENT	CATALOGUE NUMBER
**MILLIPLEX MAP Human TH17 Magnetic Bead Panel - Immunology Multiplex Assay**	**Millipore – HTH17MAG-14K**
**Dynabeads™ Untouched™ Human CD8 T Cells Kit**	**Invitrogen- 11348D**
**BD FACS ™ FACS Lysing Solution**	**BD Biosciences- 349202**
**Dynabeads™ Human T-Activator CD3/CD28 for T Cell Expansion and Activation**	**Gibco- 11132D**
**Human TruStain FcX™ (Fc Receptor Blocking Solution)**	**Biolegend- 422302**
**BD Cytofix/Cytoperm™**	**BD Biosciences- 554714**
**eBioscience™ Foxp3 / Transcription Factor Staining Buffer Set**	**Invitrogen- 11348D**
**CellROX™ Deep Red Flow Cytometry Assay Kit**	**Invitrogen- C10491**
**MitoTracker™ Red CMXRos Dye, for flow cytometry**	**Invitrogen- M46752**
**Menadione**	**Sigma Aldrich M9429**
***N*-Acetyl-L-cysteine (NAC)**	**Sigma Aldrich A9165**
**Anti-Human CD3 BV510**	**BD Biosciences- 740187**
**Anti-Human CD4 PECy7**	**BD Biosciences- 557852**
**Anti-Human CD8 AF700/BUV395**	**BD Biosciences- 561453/ 563795**
**Anti-Human CD278 (ICOS) PE**	**BD Biosciences- 557802**
**Anti-Human CXCR5(CD185) AF647**	**BD Biosciences- 558113**
**Zombie Fixable Viability™ Sampler Kit**	**Biolegend- 423117**
**Anti-Human pSTAT3 PE**	**BD Biosciences- 562072**
**Anti-Human pSTAT4 APC**	**Invitrogen- 17-9044-42**
**Anti-Human p-mTOR PECy7**	**Invitrogen- 25-9718-42**

**Anti-Human IFN-γ BV650/PECy7/AF-647**	**Biolegend/Invitrogen 502537/ 25-7319-41**
**Anti-Human IL-10 PE/BV 421**	**Invitrogen/Biolegend- 12-7108-82/ 501421**
**Anti-Human TNF-α BUV395/AF700**	**BD Biosciences- 563996/ 561023**
**Anti-Human Eomesdermin PECF594**	**BD Biosciences- 567167**
**Anti-Human Granzyme B PE**	**Biolegend- 372208**
**Anti-Human Perforin C AF594**	**Biolegend- 308124**
**Anti-Human T-bet PECy7**	**Invitrogen- 25-5825-82**
**Anti-Human Foxp3 PECy7/R718**	**Invitrogen/ BD Biosciences- 25-4777-42/ 566936**
**Anti-Human CD45 RO BV 605**	**BD Biosciences- 562791**
**Anti-Human CD45 RA BUV 496**	**BD Biosciences- 750258**
**FlowJO Version 10.8**	**BD Biosciences**
**Graph Pad Prism 9**	**Dotmatics Pvt Ltd.**

.

Table S1: Reagents and software used in the study
